# Effect of Bottlebrush Particle Architecture on Their Efficiency as Protective Layers in Li-Metal Batteries

**DOI:** 10.1021/acsapm.5c02856

**Published:** 2025-10-06

**Authors:** Verena Kempkes, Tong Liu, Jirameth Tarnsangpradit, Sipei Li, Michael R. Bockstaller, Jay F. Whitacre, Krzysztof Matyjaszewski

**Affiliations:** † Department of Chemistry, 6612 Carnegie Mellon University, 4400 Fifth Avenue, Pittsburgh, Pennsylvania 15213, United States; ‡ Department of Materials Science and Engineering, Carnegie Mellon University, 5000 Forbes Avenue, Pittsburgh, Pennsylvania 15213, United States

**Keywords:** lithium-metal battery, bottlebrush particles, artificial solid electrolyte interface, poly(ethylene oxide)-based materials, atom transfer radical polymerization

## Abstract

Due to their impressive energy density, lithium-metal batteries have great potential to replace the widely used lithium-ion batteries. However, some challenges, like dendrite growth and the formation of “dead” lithium, still hamper their industrial application to this day. Artificial solid electrolyte interfaces (aSEIs) provide a protective coating on lithium electrodes to enforce uniform lithium stripping/plating. Especially, poly­(ethylene glycol)-based polymers are promising due to their high ionic conductivity, but a challenge remains in their lack of mechanical strength. By grafting these polymers from inorganic nanoparticles, such as silica, the high ionic conductivity can be combined with the mechanical strength of the nanoparticles. The physicochemical properties of these bottlebrush particles (BBPs) are determined by the grafting density (GD), inorganic fraction, and molecular mass of the grafted polymer chains. Each of these parameters has a significant influence on the materials’ mechanical and conducting properties. In this work, each of these parameters and their effect on performance as aSEIs for lithium-metal anodes were studied. While high-molecular mass and grafting densities with low inorganic fractions can increase ion conductivity, as well as the mechanical strength of the material, unwanted elevated polarization was observed. Conversely, low GD samples provided significant enhancement of mechanical strength but reduced ionic conductivity. Overall, intermediate GD of 0.23 chains nm^–2^, high inorganic fraction of ca. 10 wt %, and intermediate molecular mass of ca. 100,000 g mol^–1^ provided the optimal balance for the tested BBPs of ionic conductivity, mechanical properties to prevent dendrite formation, as well as reduced polarization.

## Graphical abstract

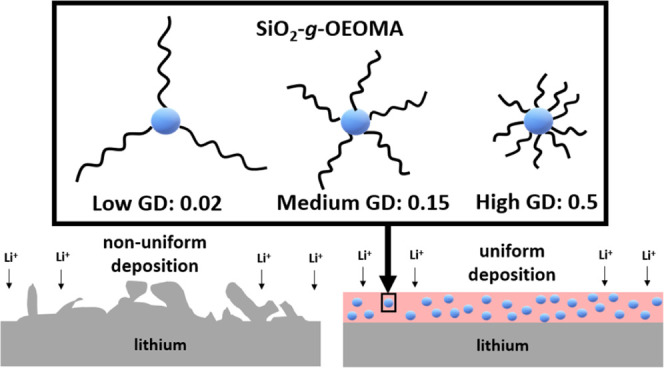

## Introduction

Since their discovery in the 1960s lithium-ion functional battery materials have been investigated, utilized, and significantly improved. With high energy density of up to 800 Wh L^–1^ ^1^ and specific energy of 200 Wh kg^–1^, as well as long cycle life of over 2000 cycles and low cost of less than 160 US$ kWh^–1^,^2^ lithium-ion batteries have been state-of-the-art for the past 30 years and are used in electric vehicles and portable electronics like mobile phones and laptops.^3^ However, the constantly evolving technology is accompanied by an ever increasing need for superior energy storage systems.^4^ The sparse storage capacity of one lithium per C_6_ of commercially used graphite anodes in combination with its relatively low density results in limited theoretical capacity of 372 mAh g^–1^.^5^ Hence, lithium-ion batteries have reached a bottleneck and are highly unlikely to be able to keep pace with the constantly rising energy demand of our society.^6–8^ Exchanging the anode material to lithium metal increases the theoretical capacity as well as the energy density to 3860 mAh g^–1^ and ∼400 Wh kg^–1^, respectively.^6,9–11^ Lithium-metal batteries, however, exhibit some challenges diminishing their promising properties caused by interfacial instabilities.^12,13^

During the charge and discharge processes, a large volume of lithium is transported from one electrode to another.^14,15^ Due to this volume change of a lithium-metal anode during cycling as well as unstable electrodeposition, lithium can be deposited in “hotspots” rather than homogeneously throughout the surface of the electrode.^16^ In multiple cycles, these “hotspots” can then grow further into lithium dendrites that cause electronic short circuits within the battery.^17^ Additionally, due to its high reactivity, lithium metal can participate in side reactions with the liquid electrolyte forming a passive solid electrolyte interface (SEI).^18,19^ This passivation of the top layer of the lithium anode leads to a significant increase in the resistance within the cell as well as the formation of lithium which is no longer connected to the current collector.^20^ Here, nonuniform lithium growth can break off of the lithium anode. These disconnected lithium pieces will passivate on the entirety of their surfaces and will not reconnect with the lithium anode itself. Hence, this so-called “dead” lithium is no longer available for energy storage, decreasing the cell’s Coulombic efficiency.^21,22^

Solid-state electrolytes have the potential to address these challenges.^23–25^ They can consist of inorganic compounds or a polymeric material. While inorganics can exhibit high ionic conductivity and mechanical strength, they commonly display brittleness.^26,27^ Hence, cell assembly and volume changes during the cycling process can cause cracking and subsequent dendrite formation due to the inflicted mechanical stress.^28,29^ Additionally, the poor interfacial contact between the electrolyte and the lithium anode leads to significantly hampered cell performances.^26^ On the other hand, polymeric materials can improve the interfacial contact to the lithium anode, while the usually low ionic conductivity leads to poorer lithium transport during cycling.^30,31^

To address this challenge, a thin layer of the functional material can be applied to the lithium metal anode as a so-called artificial solid electrolyte interface (aSEI).^32–34^ A variety of materials, inorganics as well as organics, have been used as aSEIs. While inorganics show similar advantages to the aforementioned solid-state electrolytes, the brittleness can also lead to lithium dendrite formation.^26^ In contrast, polymers feature a high level of tunability of their mechanical properties by variation of available monomer types and thus are excellent candidates for aSEI materials.^35–37^

Poly­(ethylene oxide) (PEO)-based materials, specifically, have attracted interest due to their comparatively high ionic conductivity.^24,38^ However, the poor mechanical stability caused by a low glass transition temperature (∼−60 °C) as well as its semicrystalline structure reducing ion transport limit its potential.^39,40^ One strategy to overcome these challenges is the addition of inorganic fillers.^41–43^ Grotkopp et al. incorporated silica nanoparticles into high molecular mass PEO to obtain freestanding films. This led to lithium–sulfur batteries with improved capacity retention after 350 cycles.^44^ However, the simple addition of fillers usually leads to the aggregation of the inorganic material due to the limited compatibility with the polymer matrix.^45,46^ Aggregation limits ionic transport pathways and promotes the growth of lithium dendrites. One strategy to improve compatibility and, therefore, avoid aggregation is to functionalize the nanoparticle surface. Li et al. modified silicon carbide nanoparticles using 3-(trimethoxysilyl)­propyl acylate. Mixing these functionalized nanoparticles with the PEO matrix achieved dendrite-free cycling over 200 cycles at 1C.^47^ Additionally, to ensure a uniform distribution of the silica nanoparticles, polymers were grafted from inorganic nanoparticles.^35^ Azizi et al. showed that grafting polymers, like sulfonate styrene and methyl methacrylate, from silica nanoparticles improved the compatibility with the polymer matrix within the gel polymer electrolyte.^48^ Polymer-grafted nanoparticles feature a uniform microstructure, which has been shown to promote a wide range of properties as well as suppressed aggregation. Accordingly, polymer-grafted nanoparticles have been pursued for a wide variety of applications such as lubricants,^49^ drug delivery,^50^ luminescent quantum dot materials.^51^ Unfortunately, PEO is not directly amenable to reversible deactivation radical polymerization, which is the primary method to synthesize polymer-grafted nanoparticles materials. Thus, to extend the brush concept to aSEI, we used oligo­(ethylene oxide) methyl ether methacrylate (OEOMA) as macromonomers. Due to the long side chains with an average degree of polymerization (DP) of PEO, DP = 9, the resulting polymer can be considered as a bottlebrush. Hence, the resulting bottlebrush particles (BBPs) feature a dense brush architecture and were shown to be promising candidates for aSEI in lithium–metal batteries.^52^ BBPs are defined by three parameters: molecular mass of the polymer chains (*M*_n_), grafting density (GD), and inorganic fraction (*f*_inorg_). These characteristic parameters are interconnected via the following eq 1

1 $$GD=\frac{\left(1-f_{inorg}\right)N_{A}\rho d}{6f_{inorg}M_{n}}$$

*N*_A_: Avogadro constant; ρ: density of silica nanoparticles (2.2 g cm^–3^); *d*: average diameter of silica nanoparticles (15.8 nm).

Each of the parameters affects the mechanical as well as the conductive properties of a protective layer consisting of BBPs. Since these properties are essential for the materials used as aSEIs, it is crucial to consider the effects of each of these parameters on their performance.^53^

This work aims to study the effect of architecture and composition of BBPs such as *M*_n_, GD, and *f*_inorg_ on the properties of SiO_2_-*g*-OEOMA_500_. By grafting of OEOMA_500_ from silica nanoparticles with a diameter of 15 nm, targeting different GDs and degrees of polymerization, a variety of BBP samples were synthesized with systematically varied molecular architecture. Sufficient ionic conductivity required higher GD and higher molar masses. GDs of ∼0.5–0.6 nm^–2^ are the highest achievable values due to the bulkiness of OEOMA_500_. Therefore, samples with GD values from 0.02 nm^–2^ to 0.5–0.6 nm^–2^ and molar masses above 50,000 g mol^–1^ were prepared. Mechanical properties were tested to determine the ability of BBPs to suppress lithium dendrite formation. Ionic conductivity measurements as well as testing in BBP@Li|BBP@Li symmetric cells and BBP@Li|lithium iron phosphate (LFP) half-cells were performed. Improved cycle life and low overpotentials during symmetric cycling were observed for the BBP protective layer with the best balance between all tested parameters.

## Results and Discussion

### BBP Synthesis and Characterization

To study the effect of *M*_n_, GD, and *f*_inorg_, a variety of BBPs with targeted GD and *M*_n_ were synthesized. Materials will be named using the following format: BBP_GD‑*M*_n_‑*f*_inorg__. Different GDs were achieved by functionalizing silica nanoparticles (NPs, average core diameter = 15.8 nm) with different ratios of 3-(chlorodimethylsilyl)­propyl α-bromoisobutyrate as the active ATRP initiator and chlorodimethylsilane as the inactive “dummy” initiator (Figure 1a). These “dummy” initiators cannot initiate polymerization and their incorporation leads to a decrease in the surface area functionalized with a capable initiator. The resulting GD after polymerization of these NPs is therefore reduced. Oligo­(ethylene oxide) methyl ether methacrylate (average molecular mass = 500 g mol^–1^, OEOMA_500_) was grafted from the functionalized nanoparticles via activators regenerated by electron transfer ATRP.^54^ The highest achievable GD with no added “dummy” initiator for monomers with large side-chain group—like OEOMA_500_—is 0.5–0.6 nm^–2^ which was measured for materials BBP_0.59–65k–8.4_, BBP_0.49–162k–4.2_, and BBP_0.49–297k–2.3_. By mixing active and “dummy” initiator in a 1:1 ratio, intermediate GDs of ∼0.25 nm^–2^ was obtained in BBP_0.31–135k–7.8_ and BBP_0.23–115k–11.7_. Lastly, NPs were functionalized with an active-to-dummy initiator ratio of 1:19 to synthesize BBP_0.02–454k–32.9_ and BBP_0.02–906k–17.4_ with low GD of 0.02 nm^–2^ (see Table 1).

**Figure 1:**
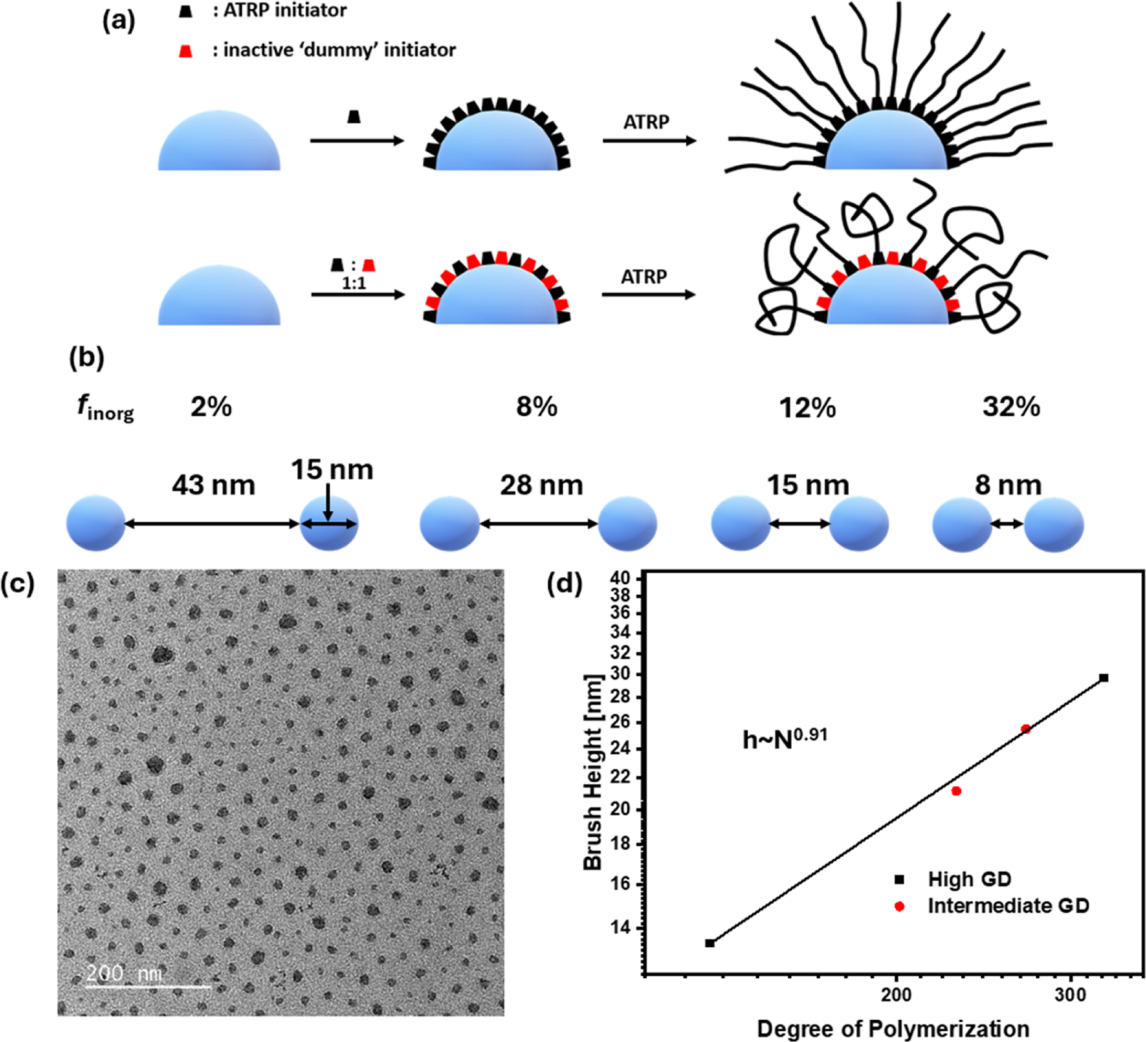
(a) Schematic illustration of lowering GD by adding “dummy” initiator, (b) schematic illustration of particle distance depending on *f*_inorg_ assuming face-centered cubic structure, (c) TEM image of BBP_0.59–65k–8.4_ after drop casting on carbon-coated copper grid and annealing at 120 °C overnight representative of high and intermediate GD samples, and (d) dependence of brush height on DP.

**Table 1:** Molecular Properties of SiO_2_-*g*-OEOMA_500_ BBPs

sample	GD_abs_ ^a^	[*M*]/[*I*]	*M*_n,app_ ^b^	*M*_n,abs_ ^c^	*D̵*	*f*_inorg_ ^d^	N_PC_ ^e^
BBP_0.59–65k–8.4_	0.59	1000/1	52,200	64,700	1.42	8.4	460
BBP_0.49–162k–4.2_	0.49	3000/1	111,400	162,000	1.58	4.2	380
BBP_0.49–297k–2.3_	0.49	5000/1	183,700	297,000	1.49	2.3	380
BBP_0.31–135k–7.8_	0.31	1500/1	95,900	135,100	1.39	7.8	240
BBP_0.23–115k–11.7_	0.23	1000/1	83,900	114,900	1.37	11.7	180
BBP_0.02–454k–32.9_	0.02	2000/1	260,500	453,500	1.37	32.9	20
BBP_0.02–906k–17.4_	0.02	5000/1	461,000	905,800	1.43	17.4	20

a Calculated by eq 1 using *M*_n,abs_.

b Determined via gel permeation chromatography in DMF at 50 °C, calibrated using poly­(methyl methacrylate) standards.

c Calculated with Mark–Houwink parameters determined in the previous report.^55^

d Determined via TGA.

e Number of polymer chains per NP.

With a GD of ∼0.5 nm^–2^, we expect that about 400 polymer chains are anchored on the NP surface (∼780 nm^2^). Due to this large amount as well as the bulkiness of OEOMA as the monomer itself, the polymer chains encounter limited space close to the nanoparticle surface. In this so-called “concentrated polymer brush regime” (CPB), the polymer chains are highly extended and have restricted chain mobility. With increasing distance from the nanoparticle surface, the available volume for the polymer chains increases, eventually allowing chains to adapt their relaxed coil conformation in the “semidilute polymer brush regime” (SDPB).^56^ Decreasing the GD to ∼0.25 nm^–2^ reduces the number of polymer chains per NP to ∼200. It allows a greater volume of polymer chains to be available closer to the NP surface. Hence, the transition from CPB to SDPB occurs at lower degrees of polymerization, and polymer chains coil at reduced molecular masses. With a further decrease of the GD to 0.02 nm^–2^, on average only 20 polymer chains are attached to each NP. Since the surface area of nongrafted NP surface is significantly larger in these BBP samples, the polymer chains in these samples can form mushroom-like structures and coils starting from the anchoring point at the NP surface. We note that the above trends have been established for linear chain brush particles, while for BBPs, no such studies have been reported. For the bottlebrush system, due to the side-chain crowding, the CPB to SDPB transition is expected to be shifted to even lower GD depending on the space requirements of the comb chains.

*F*_inorg_ significantly impacts the properties of the resulting material because it is a measurement of the number of NPs compared to polymer repeating units. To visualize this effect, the approximate distance between NPs within the material was determined by assuming a face-centered cubic (fcc) structure of the NPs (Figure 1b). For *f*_inorg_ < 12 wt % in samples with high GD and BBP_0.31–135k–7.8_, the distance between NPs exceeds the NP diameter of 15 nm while equal values of distance and NP diameter are observed for *f*_inorg_ = 12 wt % in BBP_0.23–115k–11.7_. With a further increase of *f*_inorg_ for BBP_0.02–454k–32.9_ and BBP_0.02–906–17.4_, the NP distances decrease to values below the NP diameter.

The particle spacing of the BBP samples was determined through transmission electron microscopy (TEM) images (Figures 1c and S1a–c). High as well as intermediate GD samples showed uniform spacing of the silica nanoparticles, and their interparticle distances as well as brush heights were determined (Table S1). Low GD samples, however, formed string-like aggregate structures and particle distances could not be determined (Figure S1d,e).^57^ While the interparticle distance decreased with increasing *f*_inorg_, the observed distances were significantly higher than predicted due to the assumed fcc structure. The dependence of brush height on the DP demonstrates a linear correlation for BBP samples with DP < 400 (Figure 1d). The scaling factor determined from the slope has been shown to reveal information about the conformation of polymer chains grafted from the nanoparticles. A scaling factor of 0.91 indicates that the polymer chains are within the CPB regime and, therefore, highly extended. It is important to note that previous studies have been conducted using monomers with significantly smaller functional groups, such as methyl methacrylate. This work focused on grafted dense bottlebrush-like polymers, which is a novel system that has not been extensively studied to this day.

By systematically selecting the monomer-to-initiator ratio, as well as functionalization rates of the silica nanoparticles, BBPs with varying grafting densities and molecular masses were synthesized. Gel permeation chromatography (GPC) traces in *N*,*N*-dimethylformamide (DMF) showed monomodality as well as narrow molecular-weight distributions (Figure S2).

### Mechanical Testing

One of the most important properties of materials used as aSEIs in lithium-metal batteries is the mechanical strength required to prevent the formation of lithium dendrites. The mechanical properties of the synthesized BBP samples were tested via rheological measurements (Figures 2a,b and S3).

**Figure 2:**
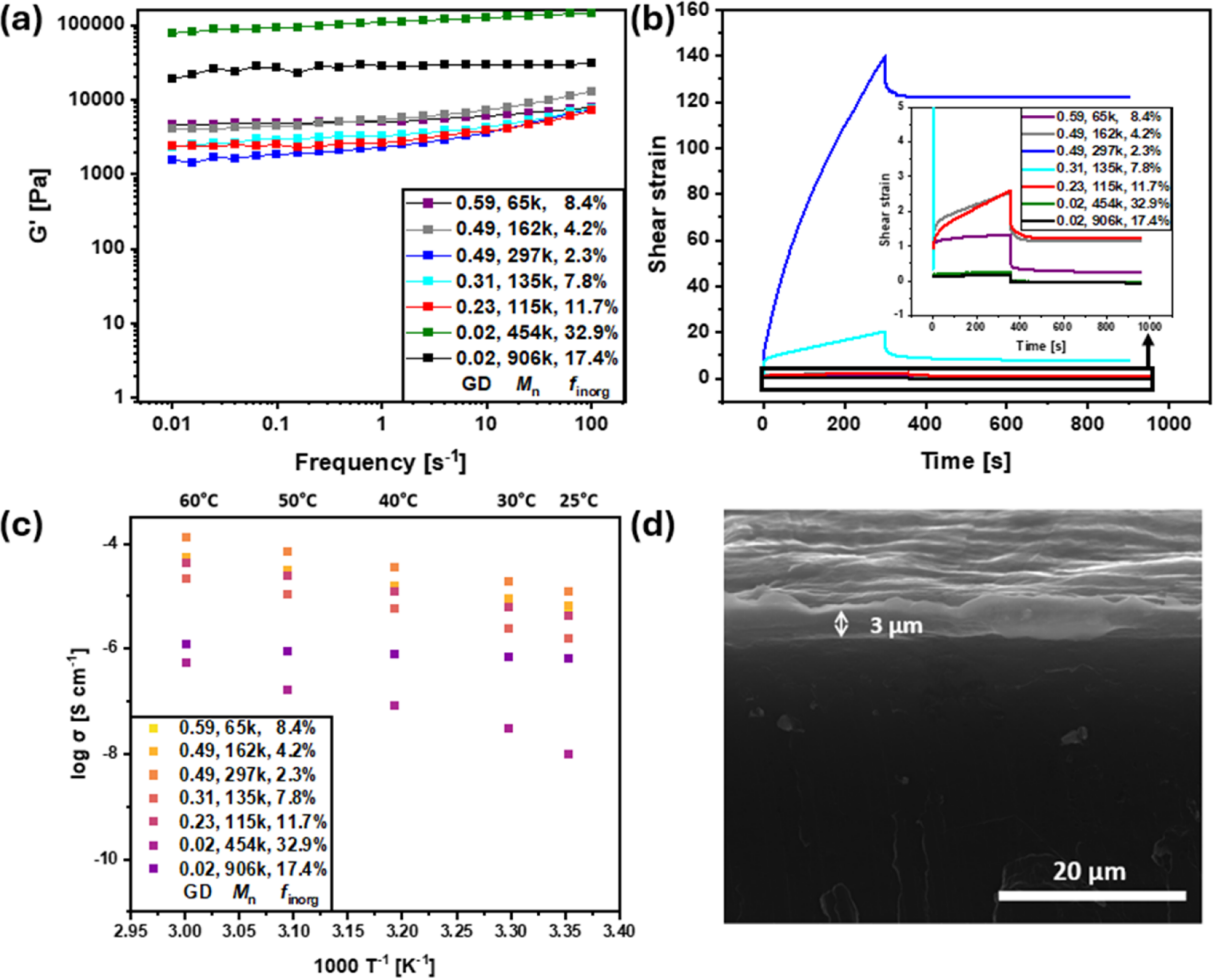
(a) Storage moduli from frequency sweeps from 0.01 to 100 s^–1^ of BBP materials at room temperature, (b) creep tests of BBP materials by applying shear stress of 50 Pa for 300 s and 0 Pa for 600 s subsequently at room temperature, (c) ionic conductivity of BBP materials mixed with LiTFSI ([EO]/[Li] = 10/1), and (d) SEM image of the cross-section of freshly drop cast BBP@Li.

The storage (*G*′) and loss moduli (*G*″) were measured using a dynamic mechanical analyzer in the frequency sweep mode from 0.01 to 100 s^–1^ (Figures 2a and S3). The highest values for *G*′ and *G*″ can be observed for BBP_0.02–454k–32.9_ and BBP_0.02–906k–17.4_ with the lowest grafting densities of 0.02 nm^–2^ and the highest inorganic fractions of 32.9 and 17.4 wt %, respectively. This significant increase in mechanical strength can be attributed to the combination of a high inorganic fraction as well as the potential aggregation of BBPs with low grafting densities into “string-like” structures. For example, Lee et al. reported a similar increase of the elastic modulus in sparsely grafted polystyrene BBPs.^53^ This aggregation results in an increase in mechanical properties. The remaining samples showed similar behaviors compared to each other, indicating that the mechanical properties are not dependent on the molecular mass of the BBP samples. Additionally, BBPs with high and intermediate grafting densities show negligible differences.

Creep tests were carried out to analyze the time-dependent mechanical properties of the BBP samples, which are important to support the large volume change of the lithium anode during the cycling process (Figure 2b). The high mechanical strength and low elasticity of BBP_0.02–454k–32.9_ and BBP_0.02–906k–17.4_ relate to the minimal values for the maximum shear strains of 0.25% and 0.16%, respectively. This indicates that these aSEIs might not be able to withstand the volume changes. With increasing values for molecular mass and GD, the shear strain and, therefore, the elasticity of the samples increase. While BBP_0.59–65k–8.4_ showed low elasticity with a maximum shear strain of 1.33%, higher molecular mass at grafting densities of 0.49 nm^–2^ as well as ∼0.25 nm^–2^ led to an increase in elasticity with maximum shear strains of 2.56%, 2.60%, and 20.5% for BBP_0.49–162k–4.2_, BBP_0.31–135k–7.8_, and BBP_0.23–115k–11.7_, respectively. The maximum shear strain of 140% for BBP_0.49–297k–2.3_ with the high GD of 0.49 nm^–2^ and molecular mass of 297,000 g mol^–1^ signifies the high contingency of this material withstanding the rapid and repeated volume change of the lithium anode. All samples showed that most of the applied deformation is permanent, which was observed for repeated cycling (Figure S4).

### Ionic Conductivity

Ionic conductivity of each BBP sample was determined via electrochemical impedance spectroscopy (EIS, Figure 2c). Inorganic fractions appear to be the main influence on the ionic conductivity of the resulting BBP materials. BBP_0.49–297k–2.3_ with the lowest inorganic fraction of 2.3 wt % showed the highest measured ionic conductivity with 14.7 × 10^–6^ S cm^–1^ at room temperature. With increasing inorganic fraction and therefore increasing content of nonconductive silica, the ionic conductivity decreases with BBP_0.02–454k–32.9_ showing a value of 5.0 × 10^–8^ S cm^–1^.

### Symmetric Li|Li Cells

BBPs were drop cast from a THF solution (100 μL of a 50 mg mL^–1^) on lithium-metal chips with a diameter of 13 mm. THF was chosen as a nonreactive solvent with a low boiling point to allow full removal of the solvent during 3 h at 65 °C. The thickness of these aSEIs was determined by scanning electron microscopy (SEM) of the coated lithium cross-section seen in Figure 2d. A thickness of 3 μm was observed for the drop cast BBP layer of 5 mg.

Symmetric BBP@Li|BBP@Li cells were assembled to study the ability of each of the BBP materials to facilitate homogeneous lithium deposition during cycling. These cells were cycled at 1 mA cm^–2^/1 mAh cm^–2^ over 1000 cycles (Figure 3a,b). The lithium anode without a protective aSEI layer showed a low overpotential of ∼30 mV for 1000 h. After this time, however, the overpotential increased rapidly, followed by a failure via short circuit at ∼1700 h. The sudden voltage increase suggests passivation of the lithium anode surface due to the formation of the natural SEI through side reactions with the liquid electrolyte.

**Figure 3:**
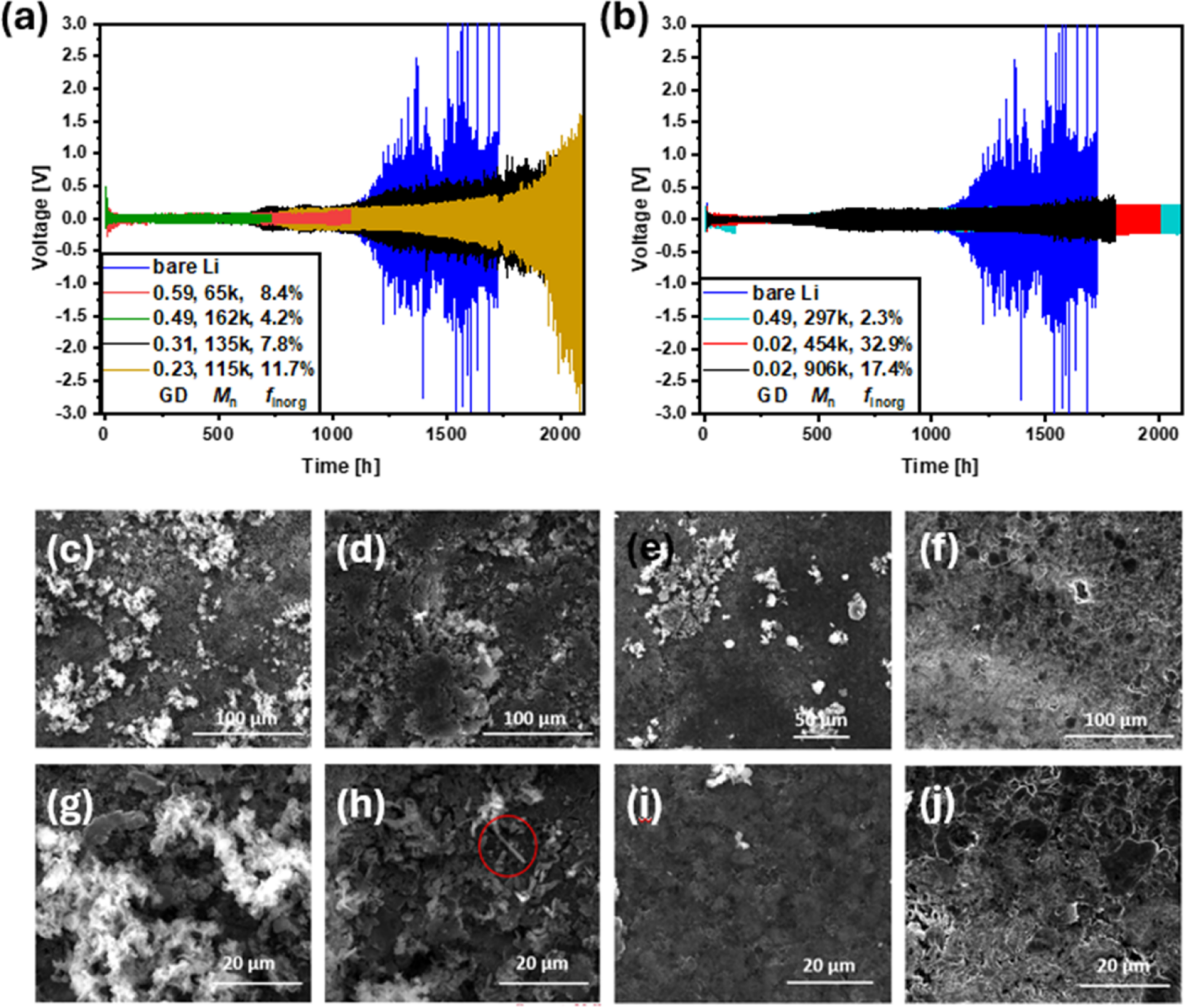
(a,b) Symmetric cycling of BBP@Li|BBP@Li cells at 1 mA cm^–2^ and 1 mAh cm^–2^ and (c–j) SEM images of lithium anode surfaces after symmetric cycling ((c) and (g) bare lithium, (d) and (h) BBP_0.49–162k–4.2_, (e) and (i) BBP_0.49–297k–2.3_, and (f) and (j) BBP_0.02–454k–32.9_).

When BBP_0.59–65k–8.4_ and BBP_0.49–162k–4.2_ are applied as aSEIs, low overpotentials were observed at low cycles. However, at ∼1100 h for BBP_0.59–65k–8.4_ and ∼750 h for BBP_0.49–162k–4.2_, the symmetric cells also failed via short circuit. BBP_0.31–135k–7.8_ and BBP_0.23–115k–11.7_ with intermediate molecular masses and lower grafting densities exceeded the cycle life of unprotected lithium. Coin cells with both aSEIs showed slightly higher overpotentials than unprotected lithium until 1000 h with 159 and 168 mV, respectively. After 1000 h, the polarization occurred in a slower manner compared to the unprotected lithium anode increasing to 2.97 V and 750 mV after the completion of cycling at 2100 h. A significant decrease in overpotentials over long cycling times was observed for the high molecular mass samples BBP_0.49–297k–2.3_, BBP_0.02–454k–32.9_, and BBP_0.02–906k–17.4_. BBP_0.49–297k–2.3_ showed an asymmetric voltage curve below 200 h, which could be attributed to inconsistencies in the protective layer caused by drop casting. However, after this occurrence, BBP_0.49–297k–2.3_ as well as BBP_0.02–454k–32.9_ showed highly symmetric cycling with low overpotentials of 225 and 218 mV after 2100 h. While low overpotentials of 200 mV were observed for BBP_0.02–906k–17.4_, the voltage curves indicated higher instability during cycling compared to BBP_0.49–297k–2.3_ and BBP_0.02–454k–32.9_.

To study the modes of failure of these cells during symmetric cycling after failure or finishing of the 1000th cycle, anode surfaces were measured via SEM (Figure 3c–j). For unprotected lithium, a notably rough anode surface was observed and attributed to nonuniform lithium deposition during cycling (Figure 3c,g). Therefore, the nonuniform growth led to spontaneous failure by penetrating the separator and connecting the electrodes, resulting in a short circuit. Similarly, BBP_0.59–65k–8.4_ and BBP_0.49–162k–4.2_ with the low molecular mass and high GD showed an irregular surface with lithium dendrites indicating nonuniform lithium deposition (Figures S5a and 2d,h). The aSEIs with intermediate grafting densities BBP_0.31–135k–7.8_ and BBP_0.23–115k–11.7_ show varying results. BBP_0.31–135k–7.8_ as aSEI resulted in unstable lithium deposition as observed by the nonuniform growths on the anode surface (Figure S5b). The anode protected by BBP_0.23–115k–11.7_, however, indicated uniform lithium deposition with a smooth surface morphology (Figure S5c). High molecular mass samples BBP_0.49–297k–2.3_, BBP_0.02–454k–32.9_, and BBP_0.02–906k–17.4_ showed significantly smoother anode surfaces compared to the previously discussed samples. The high molecular mass and GD sample BBP_0.49–297k–2.3_ presented the most homogeneous surface with small amounts of “dead” lithium (Figure 3e,i).

The lithium anode surfaces protected by BBP_0.02–454k–32.9_ and BBP_0.02–906k–17.4_ exhibited cracked surfaces (Figures 3f,j as well as S5d). This was attributed to the reduced strain-to-fracture ratio of these samples, which restricted the volume change during the cycling process and therefore promoted the formation of cracks. While the surface of unprotected lithium showed nonuniform lithium growth, no obvious lithium dendrites were detected. Lithium dendrite formation, however, was clearly observable for the low molecular mass and high GD samples BBP_0.59–65k–8.4_ and BBP_0.49–162k–4.2_. Hence, these samples do not provide the desired protection against lithium dendrite growth. Low molecular mass BBPs with high restrictions on the polymer chains can swell inhomogeneously, leading to inconsistencies within the protective layer. Subsequently, lithium is more likely to be deposited in these swollen areas due to the local increase in ionic conductivity and decrease in mechanical properties. Hence, the probability of nonuniform growth and especially dendrite formation is significantly increased as observed on the lithium anode surfaces.

BBP_0.31–135k–7.8_ and BBP_0.23–115k–11.7_ with intermediate grafting densities of 0.31 nm^–2^ and 0.23 nm^–2^ showed highly extended polymer chains as well, with a scaling factor of 0.91. However, due to the significantly lower GD, the polymer chains possessed larger free volumes closer to the nanoparticle surface. These polymer chains, therefore, had higher chain mobility and were able to swell more homogeneously. Hence, both samples were able to provide stable lithium deposition and stripping, leading to higher cycle life in symmetric cells. Due to the higher GD of BBP_0.31–115–11.7_ with 0.31 nm^–2^, the polymer chains had a lower amount of free volume and, therefore, inferior swellability, similar to the previously discussed low molecular mass and high molecular mass BBPs. Decreasing the GD with a similar molecular mass further to 0.23 nm^–2^ increased the free volume for the polymer chains. For that reason, the symmetric cell using BBP_0.23–115k–11.7_ showed cycling at low overpotential for 1750 h, while BBP_0.31–135–7.8_ experienced a significant increase after ∼1100 h.

The significantly smoother anode morphology is ascribed to the resulting increase in the mechanical strength. The lithium anode protected by BBP_0.49–297k–2.3_ showed high smoothness with occasional formation of “dead” lithium. With the higher cycle life of 2200 h, some “dead” lithium formation is expected. The smoothness of the anode surface indicated a highly homogeneous lithium deposition during the cycling process. While the lithium anode protected by BBP_0.02–454k–32.9_ as well as BBP_0.02–906k–17.4_ showed limited nonuniform growth, the surface demonstrated significant cracking. This could be related to the poor mechanical properties of the BBP materials with a low GD. Due to the low elasticity, the resulting aSEI could not support the large volume change during charge and discharge. Hence, the protective layer formed the observed cracks during the cycle life.

### Li|LFP Half-Cell Testing

Rate capability was tested in half-cells using lithium iron phosphate (LFP) as the cathode material at various current densities of 0.1 to 2 C-rate each for 5 cycles (Figure 4a). All BBP samples showed similar discharge capacities between 140 to 165 mAh g^–1^ at low C-rates of 0.1 and 0.2. With an increasing C-rate, some differences between the aSEI were observed. Low GD samples BBP_0.02–454k–32.9_ and BBP_0.02–906k–17.4_ showed the highest discharge capacities at 2 C-rate of 107.1 mAh g^–1^ and 106.7 mAh g^–1^, respectively. While values of specific discharge capacities between 60 and 95 mAh g^–1^ were determined at high C-rates, no obvious trend was observed for the residual samples with high and medium GDs. This was ascribed to small inconsistencies in the drop cast layer, which became more visible at high charge and discharge speeds. However, cells with all BBPs as aSEIs demonstrated exceptional reversibility when the low C-rate of 0.1 was applied after higher rate cycling, indicating that the protective layers could withstand higher current densities without significant degradation.

**Figure 4:**
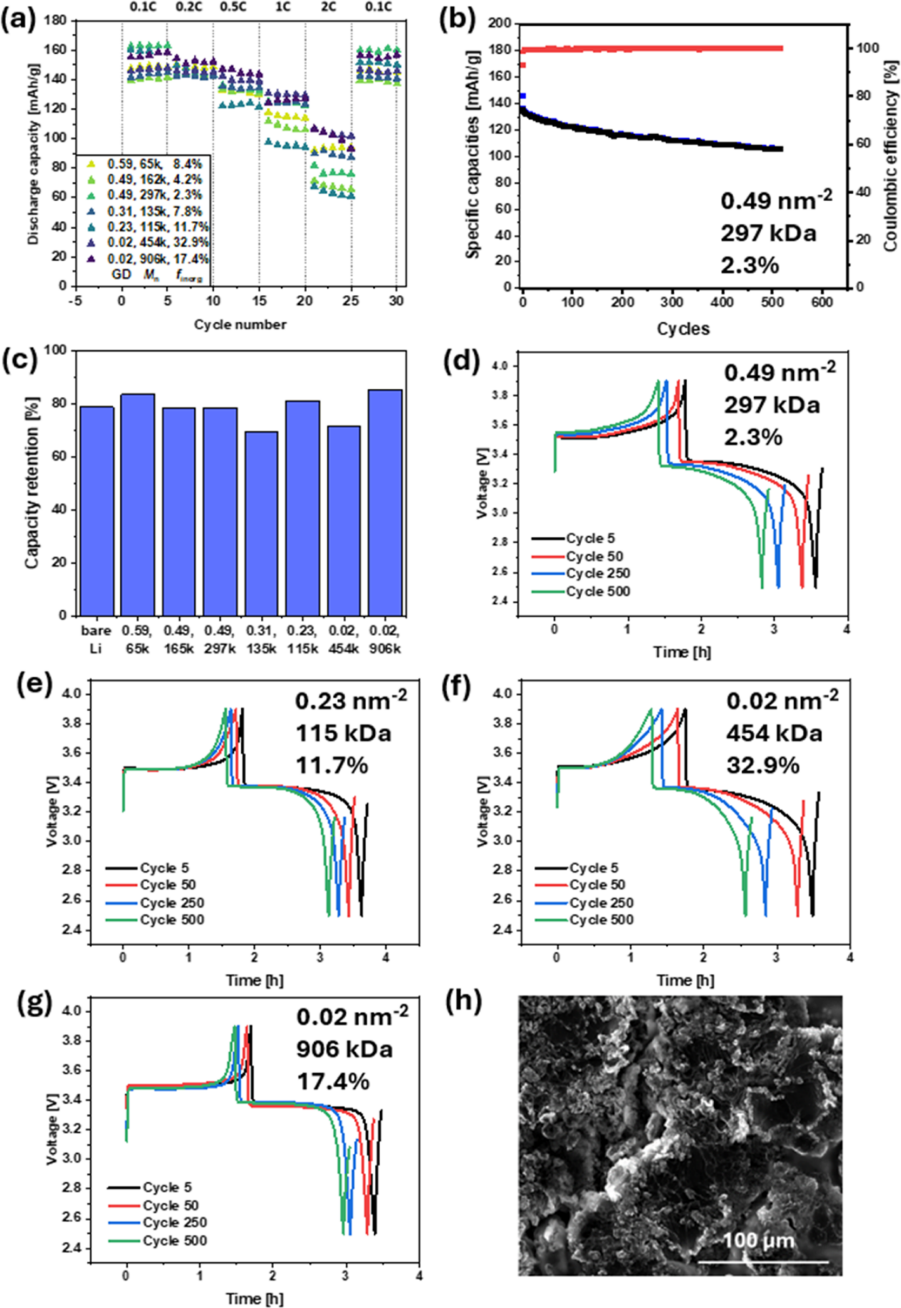
(a) Rate capability tests of BBP@Li|LFP cells, (b) cycling stability of BBP_0.49–297k–2.3_@Li|LFP cell at 0.5C, (c) capacity retention of BBP@Li|LFP cells after 550 cycles, (d–g) voltage curves of 5th, 50th, 250th, and 500th cycle of BBP@Li|LFP half-cells with a description of the structures of applied samples in each graph ((d) BBP_0.49–297k–2.3_, (e) BBP_0.23–115k–11.7_, (f) BBP_0.02–454k–32.9_, and (g) BBP_0.02–906k–17.4_), and (h) SEM image of the lithium anode protected by BBP_0.23–115k–11.7_ after 550 cycles.

Next, various aSEIs were tested in BBP@Li|LFP half-cells by cycling over 550 cycles with 0.5 C-rate (Figures 4b and S6a–f). All BBP samples showed high Coulombic efficiency of >99% with specific capacities starting at ∼135 mAh g^–1^. While specific capacities for all samples had similar behaviors, relative capacity retention was assessed in order to compare the performance of each cell (Figure 4c). With a capacity retention of 69.5% for BBP_0.31–135k–7.8_ and 71.3% for BBP_0.02–454k–32.9_, these cells showed lower retained capacity compared to that of the unprotected lithium anode with 78.8%. BBP_0.49–162k–4.2_ and BBP_0.49–297k–2.3_ demonstrated similar losses in capacities in comparison to bare lithium with 78.4% and 78.3%, respectively. An improvement in capacity retention was observed for BBP_0.59–65k–8.4_, BBP_0.23–115k–11.7_, and BBP_0.02–906k–17.4_ with 83.4%, 81.1%, and 85.3%.

To evaluate the polarization throughout the entire cycle life, the voltage curves of each BBP@Li|LFP half-cell were analyzed in their fifth, 50th, 250th, and 500th cycle (Figures 4d–h and S7). For all BBP samples, similar voltage curves were observed during their individual cycle life. Lithium anodes protected by BBP_0.59–65k–8.4_, BBP_0.23–115k–11.7_, and BBP_0.02–906k–17.4_ showed lower polarization during each cycle in their cycle lives. However, for cells in which BBP_0.49–162k–4.2_, BBP_0.49–297k–2.3_, BBP_0.31–135k–7.8_, and BBP_0.02–454k–32.9_ were used as aSEIs, the voltage curves indicated higher polarization due to the earlier voltage increase within each cycle. Cells in which BBP_0.49–162k–4.2_ were employed as the aSEI showed poor performance both in suppressing the formation of lithium dendrites and in half-cell testing. Hence, this sample was excluded from further considerations. BBP_0.49–297k–2.3_, BBP_0.31–135k–7.8_, and BBP_0.02–454k–32.9_ showed good performance in symmetric cycling due to the higher molecular mass of the grafted polymer chains. However, their higher cell polarization during cycling in half-cells also hampered their potential as aSEIs. The three aSEIs BBP_0.59–65k–8.4_, BBP_0.23–115k–11.7_, and BBP_0.02–906k–17.4_ which demonstrated lower polarization in half-cells were further considered as promising material for the protective layer. The enhanced lithium dendrite formation for BBP_0.59–65k–8.4_ observed in symmetric cells also caused this material to be excluded as a potential aSEI. While BBP_0.02–906k–17.4_ provided sufficient protection against the formation of lithium dendrites, the surface morphology of the cycled lithium anode showed some cracking caused by the low elasticity of the material. No single BBP sample showed the optimal results for the tested parameters. This signified the complexity of these materials when applied as aSEIs in lithium-metal batteries. Hence, a balance between all tested parameters was required to reveal the optimal BBP. Therefore, BBP_0.23–115k–11.7_@Li which provided uniform lithium deposition over 1000 cycles in symmetric cells in addition to the comparatively lower polarization during its half-cell cycle life was selected as the best candidate of the tested BBP samples for aSEIs.

The surface morphologies of the lithium metal anodes after 550 cycles were studied via SEM. The lithium anode surface of the BBP_0.23–115k–11.7_ material revealed the highest uniformity after cycling (Figure 4h). The residual BBP materials BBP_0.59–65k–8.4_, BBP_0.49–162k–4.2_, BBP_0.49–297k–2.3_, and BBP_0.31–135k–7.8_ (Figure S8a–d) led to significantly higher amounts of nonuniform growth and dendrite formation. On the other hand, BBP_0.02–454k–32.9_ and BBP_0.02–906k–17.4_ (Figure S8e,f) resulted in the cracking of the lithium anode surface due to the high mechanical strength of the materials.

Additionally, cross-sectional SEM images of lithium anodes coated with no BBP as well as BBP_0.23–115k–11.7_ after 80 cycles were compared (Figure S9). Here, a thinner layer of newly deposited lithium of 3.8 μm can be observed for the coated anode compared to 10.5 μm for bare lithium. This can be ascribed to a more uniform and, therefore, denser lithium deposition, showing that the protective layer led to an improved performance.

## Conclusions

In this work, the effects of molecular architecture and structural parameters of BBPs on their performance as artificial solid electrolyte interfaces in lithium-metal batteries were studied. Low GD BBP samples had a high mechanical strength caused by their low inorganic fractions and anisotropic aggregation. Ionic conductivity, however, was diminished by higher inorganic fractions along with a reduced content of nonconductive material. Cycling in symmetric Li|Li cells revealed that high molecular mass BBP samples BBP_0.49–297k–2.3_, BBP_0.02–454k–32.9_, and BBP_0.02–906k–17.4_ showed improved performance. Due to their high molecular mass, the polymer chains are within the semidilute polymer brush regime. Therefore, the enhanced performance was related to entanglements essential for protection against lithium dendrites at high cycle lives due to the increase in mechanical strength. Using lithium iron phosphate as the cathode material, the BBP were tested as aSEIs in lithium-metal anode test cells. Many samples showed excellent capacity retention and Coulombic efficiencies >99%. The voltage curves, and therefore, the development of polarization during the cycle life were compared. Here, high molecular mass samples showed increased polarization. Hence, while entanglements led to suppression of lithium dendrites, these samples showed hampered performance in half-cells. Overall, it was determined that the BBP sample with a molar mass of 114,900 g mol^–1^, GD of 0.23 chains nm^–2^ and inorganic content of 11.7 wt % showed the optimal balance for the tested BBPs between ionic conductivity, protection against lithium dendrites, and low polarization even at high cycle numbers.

## Experimental Section

### Materials

Silica nanoparticles (average core diameter *d* = 15.8 nm, 30 wt % solution in methyl ethyl ketone) from Nissan Chemicals were used as received. 3-(Chlorodimethylsilyl)­propyl α-bromoisobutyrate was synthesized via the previously reported route.^58^ Oligo­(ethylene oxide) methyl ether methacrylate with an average molecular mass of 500 g mol^–1^ (OEOMA_500_, Sigma-Aldrich) was purified by passing through basic alumina. Copper bromide (CuBr_2_, Sigma-Aldrich, 99.9%), tris­(2-dimethylaminoethyl)­amine (Me_6_TREN, Ambeed, 99%), tin­(II) 2-ethylhexanoate (Sn­(EH)_2_, Sigma-Aldrich, 95%), 48 wt % aqueous hydrofluoric acid (HF, 99.99%, Sigma-Aldrich), alumina (basic, Super I, 50–200 μm, Sorbtech), lithium bis­(trifluoromethane)­sulfonimide (LiTFSI, Sigma-Aldrich, anhydrous, 99.99%), lithium nitrate (LiNO_3_, 99.99% Sigma-Aldrich), anisole (Thermo Fisher, 99%), *N*,*N*-dimethylformamide (DMF, Thermo Fisher, 99.8%), hexane (Thermo Fisher, 98.5%), methanol (MeOH, Thermo Fisher, 99.8%), tetrahydrofuran (THF, Sigma-Aldrich, 99.5%), 1,3-dioxolane (DOL, Sigma-Aldrich, anhydrous, 99.5%), 1,2-dimethoxyethane (DME, Sigma-Aldrich, anhydrous, 99.5%), and 1-methyl-2-pyrrolidinone (NMP, Sigma-Aldrich, anhydrous, 99.5%) were used as received.

### BBP Synthesis

Silica nanoparticles were functionalized with 3-(chlorodimethylsilyl)­propyl α-bromoisobutyrate as described in a previous publication.^58^

Silica nanoparticles were dispersed in OEOMA_500_, anisole (1:1 v/v), and DMF (1 mL). CuBr_2_ (200 ppm) and Me_6_TREN ([Cu]/[ligand] = 1/3) were added. The reaction mixture was added to a clean, dry Schlenk flask. After sealing the flask, the reaction mixture was deoxygenated by sparging with nitrogen gas for 20 min. The flask was lowered into an oil bath, and Sn­(EH)_2_ ([Cu]/[Tin] = 1/5) was added dropwise. The reaction mixture was stirred at 50 °C until viscous. The BBP was purified via precipitation in hexane and further dialysis in MeOH (1 cycle) and THF (2 cycles).

### Analysis

GPC samples were prepared via etching with HF for 12 h and neutralizing with ammonia. The prepared samples were run through a 1 mL neutral alumina column and a 0.45 μm PTFE filter. Number-average molecular mass (*M*_n_) and molecular weight dispersity (*M*_w_/*M*_n_) were measured in THF as the solvent at a flow rate of 1.00 mL/min at 35 °C in an Agilent GPC using polymer standards services columns (guard, 10^5^, 10^3^ and 10^2^ Å) and a differential refractive index detector (Waters, 2410).

SEM was performed using a Quanta 600 FEG instrument to investigate the surface morphology of the lithium electrodes.

TEM was carried out using an FEI Tecnai F20 Super-Twin electron microscope operated at 200 kV to determine interparticle distance. Samples were drop cast onto a carbon-coated copper grid before measurement.

Thermogravimetric analysis (TGA) was performed using a TA Instruments 2950 instrument to measure the silica (inorganic) fraction within the BBPs. The data were analyzed using TA Universal Analysis. The heating procedure included three steps: ramp up at a rate of 30 °C/min to 120 °C, hold at 120 °C for 10 min, and ramp up at a rate of 20 °C/min to 800 °C. The inorganic fraction was calculated from the weight loss between 120 and 800 °C.

The scaling factor was approximated by plotting the brush height from the TEM images as a function of *M*_n,abs_. The TEM images are shown in Figure S3a–d. The brush height of each sample was measured by subtracting the core-to-core distance between the particles with each core radius and dividing by two. The results are summarized in Table S1 and plotted in Figure S3e. The scaling factor obtained is 0.91, indicating that the particles behave closely to a CPB regime where the grafted chains exhibit extended chain conformations.

Mechanical testing was conducted by using an Anton Paar MCR-302 Rheometer fitted with a parallel plate tool. BBPs were drop cast on the plate, and a nominal force of 1 N was applied. At room temperature, frequency sweeps were carried out from 0.01 to 100 s^–1^ to determine the storage and loss moduli. Creep tests were performed by applying a shear stress of 50 Pa for 300 s and 0 Pa for 600 s consecutively. Shear strain values were reported as percentages.

Electrochemical testing was carried out by using CR2032-type coin cells. All cells were prepared in a glovebox with water and oxygen contents of lower than 0.5 ppm and were tested at room temperature.

Ionic conductivity measurements were tested by using AC impedance spectroscopy. BBP samples were sandwiched in stainless-steel|BBP mixed with LiTFSI (EO/Li = 10:1)|stainless-steel cells. These measurements were carried out at temperatures from 25 to 60 °C, and the cells were equilibrated for 15 min at each temperature before measurements. The ionic conductivity values were calculated with the charge-transfer resistance using the following equation, in which *L* is the thickness of the BBP material, *A* is the area of the material, and *R* is the charge-transfer resistance determined from the Nyquist plots.

$$\sigma =\frac{L}{R\cdot A}$$

BBP samples (5 mg) were drop cast from a THF solution (100 μL of a 50 mg/mL) on lithium chips (13 mm diameter and 0.6 mm thickness) and dried for 3 h at 65 °C. For symmetric cycling, two BBP@Li electrodes were assembled to study the long-term electrochemical performance of Li plating/stripping at 1 mA cm^–2^ and 1 mAh cm^–2^. For Li|lithium iron phosphate (LFP) cells, the cathode was prepared by mixing 85 wt % of commercial LFP powder, 10 wt % of Super-p, and 5 wt % of polyvinylidene difluoride binder using NMP as the solvent. The slurry was cast onto Al foil with a mass loading of 1.5 mg cm^–2^. For the liquid electrolyte, 1 M LiTFSI in DOL and DME (1:1 v/v) with 2 wt % LiNO_3_ was prepared and 60 μL were used during the assembly of all cells. For rate capability testing, the discharge capacity was measured at C-rates of 0.1, 0.2, 0.5, 1, and 2 for 5 cycles each. For long-term cycling, 6 formation cycles at 0.1C were carried out with 500 cycles following at 0.5C.

## Supplementary Material



## Notes

Additional TEM and SEM images, GPC traces, frequency sweeps, creep tests, and cycling data for all samples (PDF)
