# Supplementary material for: Effect of Bottlebrush Particle Architecture on Their Efficiency as Protective Layers in Li-Metal Batteries
Source: ACS Appl Polym Mater. 2025 Oct 6;7(20):13885–95. doi: 10.1021/acsapm.5c02856 (PMC12560076; doi:10.1021/acsapm.5c02856)
Supplement: Supplementary file 1 [file ap5c02856_si_001.pdf]

Supporting Information

Effect of Bottlebrush Particle Architecture on Their Efficiency as Protective Layers in Li-Metal Batteries

Verena Kempkes[a], Tong Liu[a], Jirameth Tarnsangpradit[b], Sipei Li[a], Michael R. Bockstaller[b], Jay F. Whitacre*[b], Krzysztof Matyjaszewski*[a]

[a]Department of Chemistry
Carnegie Mellon University
4400 Fifth Avenue, Pittsburgh, PA 15213, USA
E-mail: matyjaszewski@cmu.edu

[b]Department of Materials Science and Engineering
Carnegie Mellon University
5000 Forbes Avenue, Pittsburgh, PA 15213, USA
E-mail: whitacre@andrew.cmu.edu

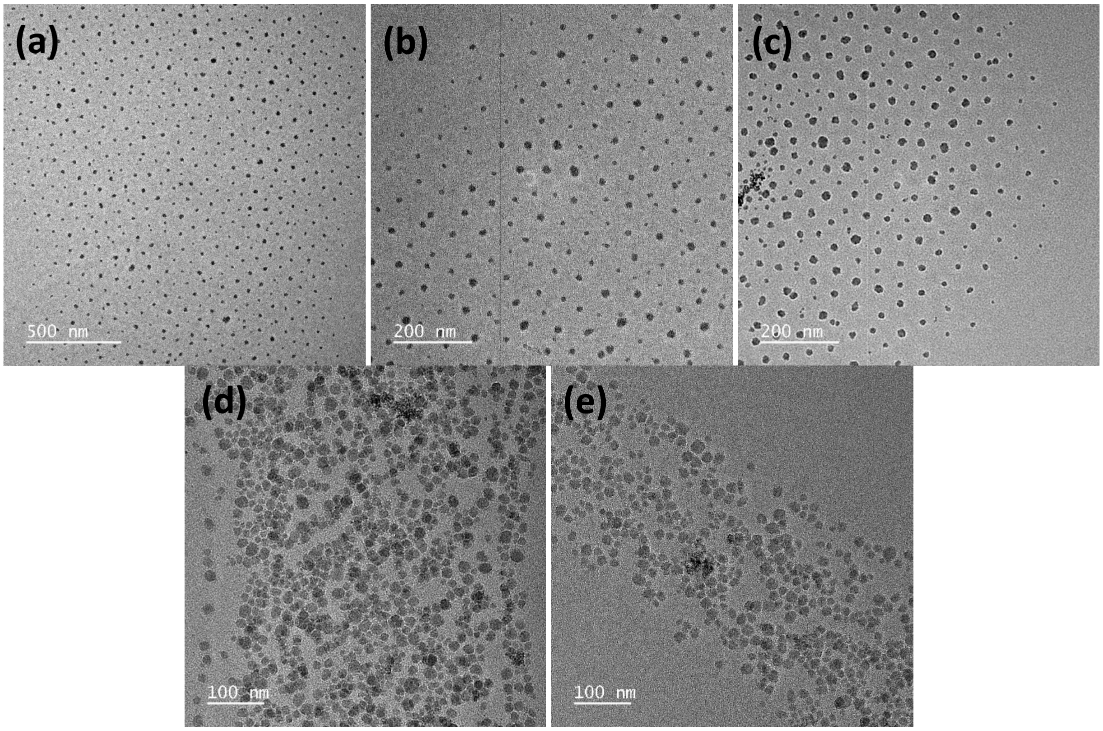

Figure S1: TEM images of (a) $BBP_{0.49\text{-}162k\text{-}4.2}$, (b) $BBP_{0.31\text{-}135k\text{-}7.8}$, (c) $BBP_{0.23\text{-}115k\text{-}11.7}$, (d) $BBP_{0.02\text{-}454k\text{-}32.9}$ and (e) $BBP_{0.02\text{-}906k\text{-}17.4}$ after casting on a carbon-coated copper grid from a THF solution and annealing under vacuum at 120°C overnight.

Table S1: Interparticle distance and brush height of BBP samples with high and intermediate GD.

Sample	Interparticle distance [nm]	Brush height, h [nm]
$BBP_{0.59\text{-}65k\text{-}8.4}$	43.10 ± 5.53	14.05 ± 2.76
$BBP_{0.49\text{-}162k\text{-}4.2}$	80.04 ± 12.11	32.52 ± 6.06
$BBP_{0.31\text{-}135k\text{-}7.8}$	66.86 ± 14.26	25.49 ± 7.52
$BBP_{0.23\text{-}115k\text{-}11.7}$	58.10 ± 13.90	21.16 ± 7.28

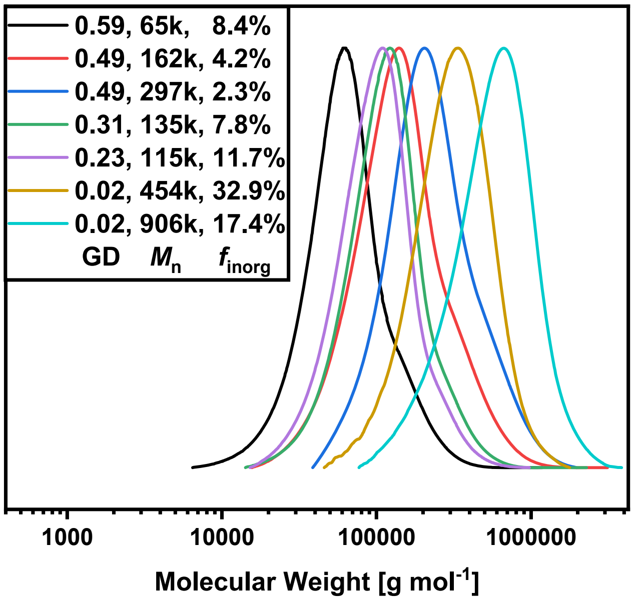

Figure S2: GPC traces of bottlebrush polymer chains after detaching from silica nanoparticles with HF.

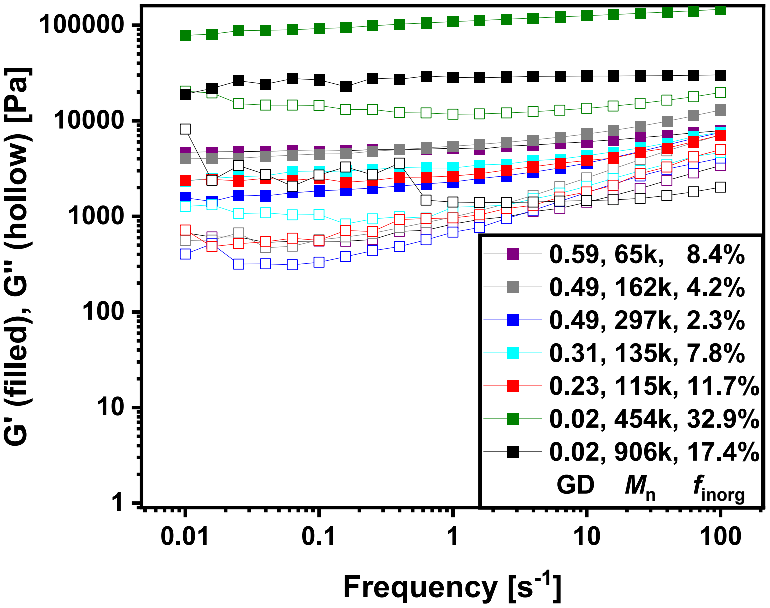

Figure S3: Frequency sweeps from 0.01 to 100 s^{-1} of BBP materials at room temperature.

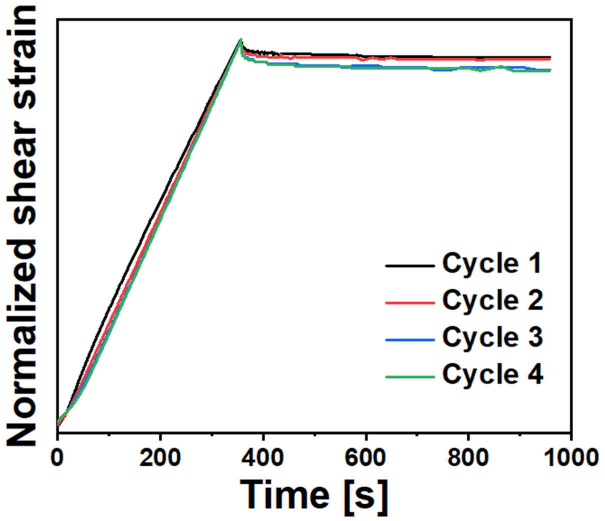

Figure S4: Repeated cycles of creep test of $BBP_{0.23\text{-}115k\text{-}11.7}$ by applying a shear stress of 50 Pa for 350 s and 0 Pa for 600 s at room temperature.

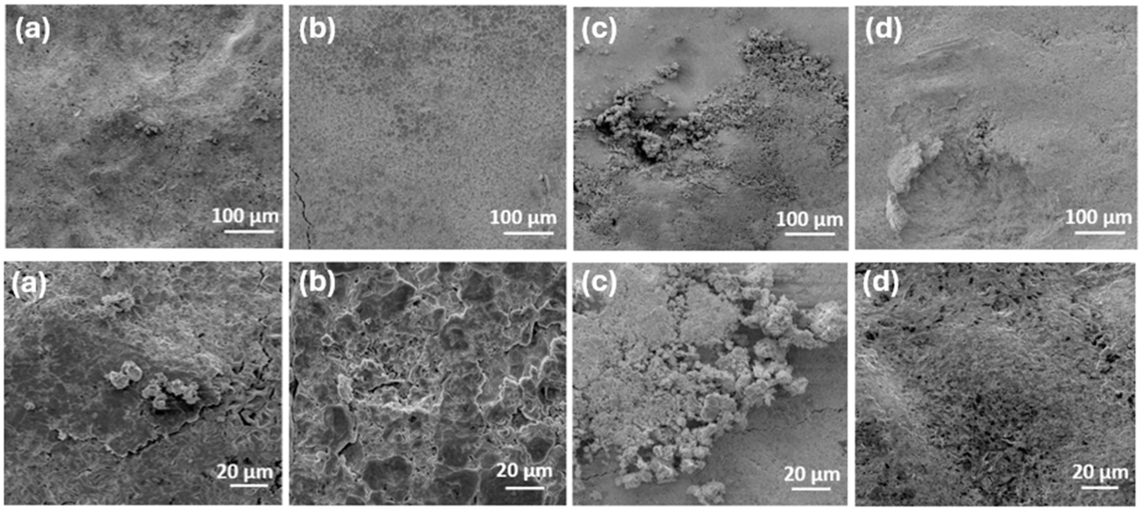

Figure S5: SEM images of Li anode surfaces after symmetric cycling ((a) $BBP_{0.59\text{-}65k\text{-}8.4}$, (b) $BBP_{0.31\text{-}135k\text{-}7.8}$, (c) $BBP_{0.23\text{-}115k\text{-}11.7}$ and (d) $BBP_{0.02\text{-}906k\text{-}17.4}$).

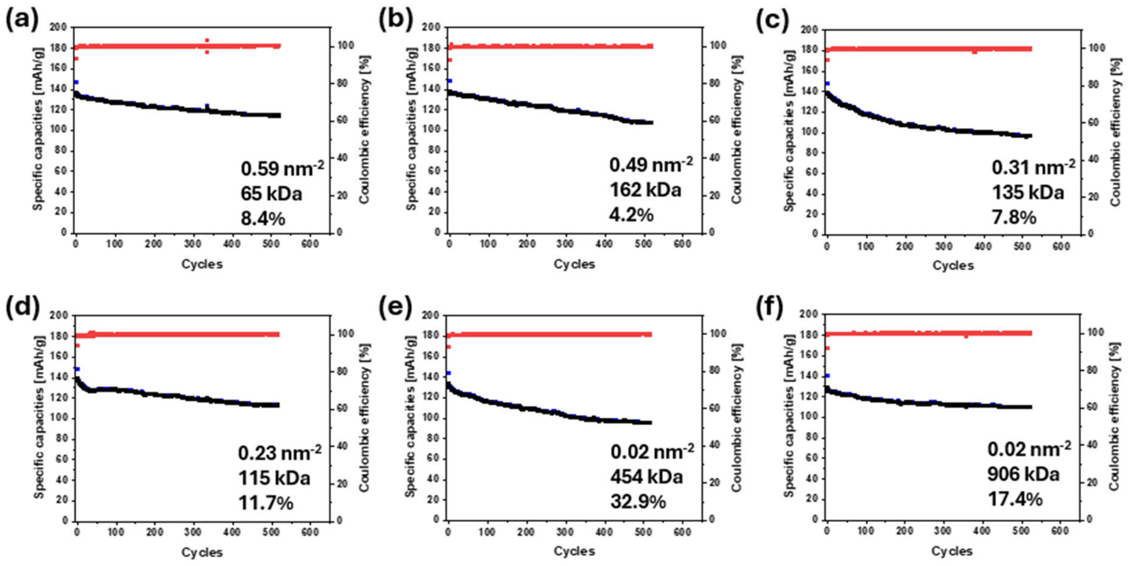

Figure S6: Cycling stability of BBP@Li|LFP cells at 0.5C ((a) BBP$_{0.59\text{-}65k\text{-}8.4}$, (b) BBP$_{0.49\text{-}162k\text{-}4.2}$, (c) BBP$_{0.31\text{-}135k\text{-}7.8}$, (d) BBP$_{0.23\text{-}115k\text{-}11.7}$, (e) BBP$_{0.02\text{-}454k\text{-}32.9}$ and (f) BBP$_{0.02\text{-}906k\text{-}17.4}$) with a description of the applied samples within each graph.

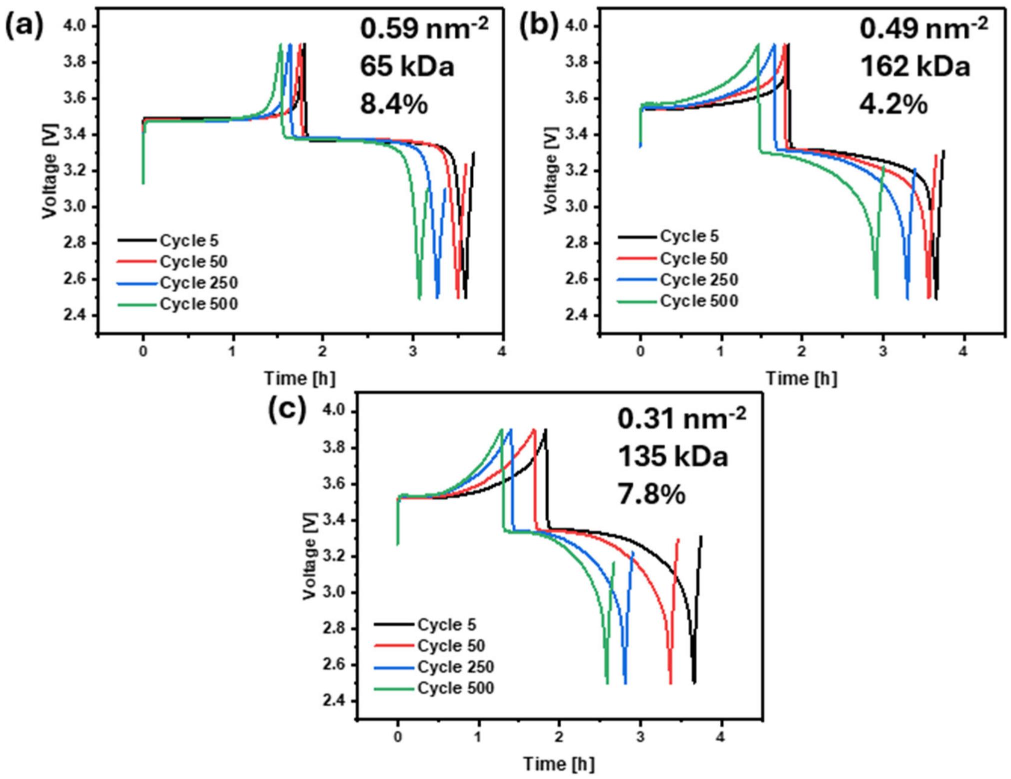

Figure S7: Voltage curves of 5th, 50th, 250th and 500th cycle of BBP@Li|LFP half cells ((a) $BBP_{0.59\text{-}65k\text{-}8.4}$, (b) $BBP_{0.49\text{-}162k\text{-}4.2}$ and (c) $BBP_{0.31\text{-}135k\text{-}7.8}$) at 0.5C with a description of the applied samples in each graph.

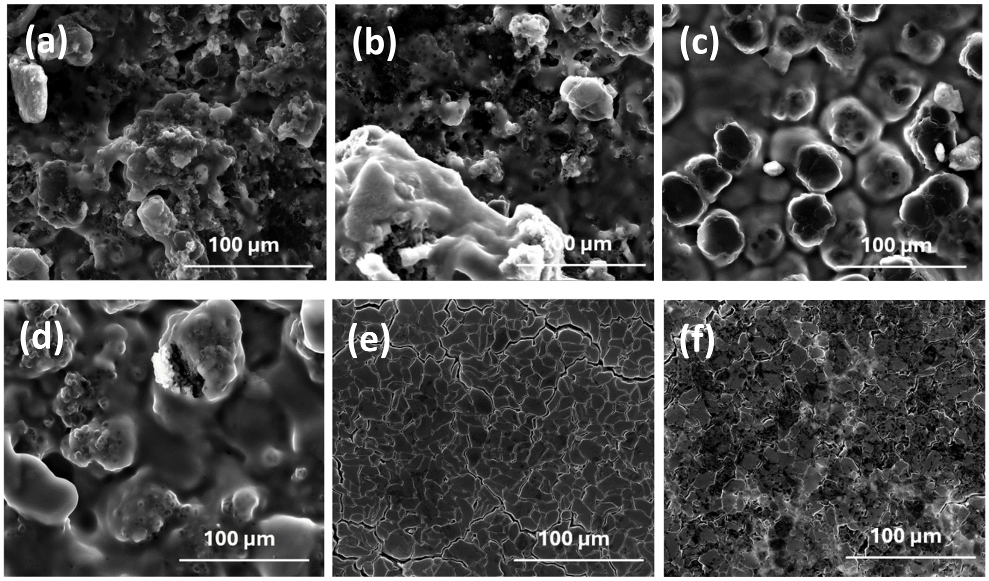

Figure S8: SEM images of Li anode protected by (a) $BBP_{0.59-65k-8.4}$, (b) $BBP_{0.49-162k-4.2}$, (c) $BBP_{0.49-297k-2.3}$, (d) $BBP_{0.31-135k-7.8}$, (e) $BBP_{0.02-454k-32.9}$ and (f) $BBP_{0.02-906k-17.4}$ after 550 cycles.

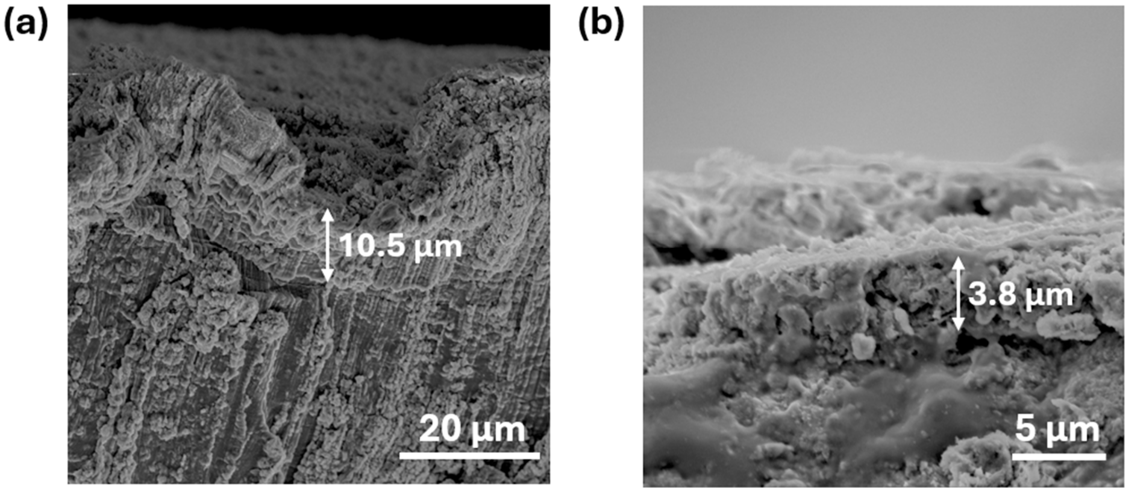

Figure S9: Cross-sectional SEM images of lithium anodes of (a) bare lithium and (b) coated with $BBP_{0.23-115k-11.7}$ after 80 cycles.
